# Perceptions of non-sugar sweeteners and front-of-package labels among parents of preschool and school-aged children in Brazil

**DOI:** 10.1017/S1368980025101146

**Published:** 2025-09-19

**Authors:** Mariana Fagundes Grilo, Beatriz Silva Nunes, Maria Fernanda Eberle, Natalie Vallone, Claudia Nieto, Uriyoán Cólon-Ramos, Lindsey Smith Taillie, Ana Clara Duran, Allison C. Sylvetsky

**Affiliations:** 1 Department of Exercise and Nutrition Sciences, Milken Institute School of Public Health, The George Washington University, Washington, DC, USA; 2 Center for Food Studies and Research, University of Campinas, Campinas, Brazil; 3 Graduate Program in Collective Health, Faculty of Medical Sciences, University of Campinas, Campinas, Brazil; 4 Faculty of Medicine, Universidad Nacional Autónoma de México, Mexico City, Mexico; 5 Department of Global Health, Milken Institute School of Public Health, The George Washington University, Washington, DC, USA; 6 Department of Nutrition, Gillings School of Global Public Health, UNC-Chapel Hill, Chapel Hill, NC, USA; 7 Carolina Population Center, University of North Carolina at Chapel Hill, Chapel Hill, NC, USA; 8 Center for Epidemiological Studies in Nutrition and Health, University of São Paulo, São Paulo, Brazil

**Keywords:** Non-sugar sweeteners, Front-of-package labelling, Policy, Children

## Abstract

**Objective:**

To describe Brazilian parents’ perceptions of non-sugar sweeteners (NSS) in beverages consumed by children and their views for NSS front-of-package labels (FOPL).

**Design:**

A qualitative-driven mixed-methods embedded design was used. Seven focus groups with parents of children explored perceptions of NSS. Qualitative data were coded and analyzed using thematic analysis. Participants also completed a closed-ended survey assessing familiarity with NSS-containing beverages, ability to identify NSS on ingredient labels and perceptions of NSS FOPL. Survey responses were summarised using descriptive statistics.

**Setting:**

Public and private schools and early childhood education centres in urban areas of two municipalities in the State of São Paulo, Brazil.

**Participants:**

Forty parents of children aged 2–5 and 6–11.

**Results:**

About 35 % of participants reported their children consumed at least one NSS-containing beverage weekly in the past month; 17 % reported daily consumption. Parents expressed a preference for natural products and confusion over the term ‘*edulcorantes*’ (Portuguese for NSS). They shared concerns about the health effects of both sugar and NSS, particularly for children. NSS were seen as acceptable in specific cases, such as if a child has diabetes. Most parents supported a FOPL like Mexico’s, stating ‘not recommended for children’. In the survey, 85 % of the parents correctly identified beverages with NSS, but 82 % misclassified non-NSS ingredients (e.g. sugar syrup, caramel) as NSS. The Mexico-style FOPL was preferred by 95 % of the parents, who found it helpful and easy to understand.

**Conclusions:**

A FOPL clearly indicating NSS presence, especially one recommending against consumption by children, may help parents make informed choices and reduce children’s intake of NSS-containing beverages.

Parents play a critical role in shaping their children’s eating habits, which have long-lasting effects on growth, development and health outcomes^(1)^. As primary gatekeepers of their children’s nutrition, parents influence food choices through purchasing decisions, meal preparation, parent modelling and providing guidance on dietary choices^(1)^. Currently, there is limited understanding of how parents perceive non-sugar sweeteners (NSS) in beverages consumed by children. NSS, also referred to as non-nutritive or artificial sweeteners, are additives used to sweeten foods and beverages without adding calories or sugar^(2)^. Common examples of NSS include aspartame, sucralose, stevia, monk fruit extract and acesulfame-potassium^(2)^. Despite their lack of calories, NSS are not metabolically inert, and recent evidence reinforces that more studies on the health impacts of NSS in all life stages are warranted, especially among children^(3)^.

The WHO advises against the use of NSS for weight management or the prevention of non-communicable diseases, citing insufficient evidence on their long-term safety, including among children^(4)^. This recommendation is particularly relevant for children, as their dietary habits and taste preferences are still developing. There are concerns that consuming NSS may cultivate a preference for sweet taste, potentially leading children to seek out more sugary foods, which could undermine efforts to promote healthier dietary patterns^(5)^. Additionally, given that children have lower body weights and are still developing, NSS exposure during childhood may have distinct physiological effects that have not been well-studied^(5)^.

Due to the adverse health effects of excessive sugar consumption, such as obesity and cardiovascular diseases^(6,7)^, many governments worldwide have implemented policies to reduce sugar intake, including front-of-package labels (FOPL). FOPL help consumers identify products high in added sugars and other nutrients of concern^(8)^ and encourage food and beverage manufacturers to reformulate their products. While these policies have been successful in reducing the sales and consumption of sugary drinks^(9,10)^, they can also lead to an increased use of NSS in the food supply^(11)^.

In Brazil, FOPL regulations introduced in 2020 mandate warnings for products high in added sugars, sodium and saturated fats, but there is currently no requirement for labelling products containing NSS^(12)^. The declaration of NSS on food packages is regulated by the Agência Nacional de Vigilância Sanitária (ANVISA) and currently requires that NSS be listed in the list of ingredients of food and beverage labels^(13)^. Similar FOPL policies that did not include an NSS FOPL in countries like Chile have resulted in an increased use of NSS in the food supply^(11)^. Products such as dairy beverages, ice creams, sodas and powdered juices are predominant sources of NSS^(14)^.

While recent data suggest that approximately 40 % of the population in a large Brazilian city consumes products containing NSS^(15)^, the attitudes and behaviours of Brazilian parents regarding NSS for their children have not been examined. Understanding Brazilian parents’ perceptions of NSS and their perceptions of FOPL communicating the presence of NSS on product packaging is important, particularly considering the recently implemented FOPL for added sugar in Brazil and considering the marked increases in the use of NSS after the implementation of similar policies. Herein, we report the findings of a qualitative-driven mixed-methods study designed to explore Brazilian parents’ and caregivers’ (hereafter parents) perceptions and knowledge surrounding NSS and NSS FOPL in beverages consumed by children.

## Methods

This study used a qualitative-driven mixed-methods embedded design that focused on the collection and analysis of qualitative data, along with a survey to explore Brazilian parents’ perceptions and understanding of NSS in children’s beverages and their perceptions of FOPL for NSS. Qualitative-driven mixed-methods embedded design is useful when quantitative data provide a supportive secondary role in a study that is primarily qualitative on the premise that a single dataset is insufficient to answer our key research questions^(16)^. The key research questions for the qualitative focus group discussions centred on parents’ understanding of ‘*edulcorantes*’, (the Portuguese term for NSS), the factors influencing their choices regarding NSS-containing beverages, their concerns about NSS in children’s diets and their reactions to different hypothetical NSS FOPL. For the quantitative survey, the key questions focused on parents’ familiarity with NSS-containing beverages, their ability to identify NSS on packaging and their perceptions of different NSS FOPL designs in terms of clarity, usefulness and influence on purchasing decisions. The order of the focus group discussions followed by the survey was chosen because the primary objective was to understand parents’ perceptions of NSS and NSS FOPL in beverages consumed by children. The study focused specifically on beverages containing NSS because they are the predominant source of NSS consumption among children and are often marketed to children^(17)^.

The study was conducted according to the guidelines in the Declaration of Helsinki, and all procedures involving research study participants were approved by the University of Campinas and National Ethics Committee (CEP/CONEP system) (70104423.1.0000.5404) and the George Washington University Institutional Review Board (NCR234740). Written informed consent was obtained from all participants prior to beginning the study procedures.

### Recruitment and eligibility

The research team contacted public and private schools and early education centres in urban areas from two municipalities of the State of São Paulo, Brazil (Campinas and Itatiba), via email and/or telephone. This approach was used because public schools in Brazil generally serve students from lower socio-economic backgrounds, while private schools are typically attended by students from higher socio-economic backgrounds^(18)^. For schools and early education centres that expressed interest in the research project, a Zoom or in-person meeting with a member of the research team was scheduled to discuss the project’s purpose. Schools and early education centres that agreed to collaborate actively participated in the recruitment process by sending parents of their students videos and messages with information on the study aims and design. Schools offered their facilities for conducting the study procedures, which minimised participant burden.

Inclusion criteria required participants to be parents of a child 2–5 years old or 6–11 years old (parents had to have a child in one of these age ranges to participate), in addition to being responsible for at least half of the child’s grocery/food shopping and living in the same home as the child. Parents who reported that they had a child with a diet-related disease or condition requiring a special diet were excluded because these parents are more likely to check nutrition information on product labels^(19)^. Focus group discussions were conducted separately based on the child’s age range, considering the differences in exposure and consumption of beverages in preschool *versus* school-aged children^(20,21)^.

### Study procedures

Focus groups were scheduled with the assistance of participating schools and early education centres and took place between July and November 2023. All participants signed a consent form prior to the study and had the opportunity to ask questions about the study design and aims.

Before data collection, the focus group guide and survey instrument were pilot tested with five parents of children aged 2–5 and 6–11 years old. Feedback from pilot testing informed minor wording revisions and modifications to the structure of the focus group guide and survey to ensure participant understanding.

### Participants’ characteristics and beverage frequency questionnaire

After providing informed consent, participants completed a sociodemographic questionnaire including questions about their age, gender, race, ethnicity, household income, highest level of education completed, marital status, household size (including the number of children under 10) and work schedule. Participants reported their child’s age (for their oldest child within the study’s target age range) and their child’s typical beverage consumption in the past month using an adapted beverage frequency questionnaire (BFQ)^(22,23)^. The questionnaire followed a structured format where participants indicated how often their child consumed each beverage over the past month using predefined categories (ranging from ‘never or less than once per week’ to ‘daily consumption’) and the approximate amount their child consumed per occasion. The response options for beverage volume varied depending on the child’s age range; the amount of beverages reported in the BFQ developed by Lora *et al*. for preschool children (2016) was used for children aged 2–5 years old^(22)^, and the amount of beverage in the BFQ developed by Headrick *et al*. (2012) was used for children aged 6–11 years old^(23)^. Participants were provided with pictures to facilitate accurate portion size reporting.

The BFQ included a variety of beverages to comprehensively capture children’s beverage consumption patterns. These included water, flavoured water, 100 % fruit juice, powdered juice mixes, ready-to-drink sweetened juices, dairy-based beverages (full-fat, semi-skimmed and skimmed milk), flavoured milk drinks (such as chocolate and strawberry milk), plant-based milk alternatives (including soy, almond and oat milk), regular and diet soft drinks, sports and energy drinks, sweetened tea, unsweetened tea, coffee with and without milk and other beverages as specified by respondents. The categories were based on the validated BFQ^(22,23)^ with some adaptations (i.e. addition of flavoured water and powdered juices) to the Brazilian cultural context^(22,23)^. The adaptation process included translating the original US questionnaire into Portuguese, converting measurements from ounces to millilitres and including local brands as examples for the beverage categories.

NSS-containing beverages included the following items on the BFQ: addition of NSS to coffee, coffee with milk and tea; artificially sweetened beverages (e.g. diet, light, no sugar, zero sugar); and powdered juice^(14)^. The category of artificially sweetened beverages (e.g. light, diet, zero) encompasses a variety of beverage types, including soft drinks, juices and other commonly consumed drinks that are explicitly marketed as low-calorie or sugar-free. These products were grouped together in the BFQ to capture a broad range of NSS-containing options under a single category. Powdered juices were also included as containing NSS, as all brands in Brazil, even those marketed as regular options, contain NSS^(14)^. Additionally, since the BFQ format did not provide access to the ingredient lists of the beverages consumed, the selected categories focused on beverages most likely to contain NSS, based on their labelling claims and common formulations in Brazil.

### Qualitative component: focus group discussions

The focus group discussions were facilitated by a nutritionist trained in qualitative data collection, along with a research assistant, and followed a semi-structured guide (see online supplementary material, Supplemental 1) developed by the research team. The guide was informed by prior studies related to NSS perceptions and consumer understanding of FOPL^(24,25)^. This approach ensured that the discussion covered key areas relevant to parents’ knowledge, attitudes and behaviours towards NSS and labelling, while also allowing for flexibility in capturing emerging themes that had not been previously documented in the literature. The guide was designed to spark a rich discussion with parents about their beverage choices for their children, their understanding of NSS and the term ‘*edulcorantes*’, how they perceived the healthfulness of NSS compared with sugars, their awareness regarding FOPL in food and beverage items in Brazil and whether and how labelling and NSS disclosures influenced their purchasing decisions. The research assistant monitored non-verbal cues, such as body language, to capture emotions during the discussions. Before posing questions specifically, such as where they can be found, parents were asked if they knew what NSS were. This served to familiarise everyone with the term; a definition was provided (see online supplementary material, Supplemental 1).

Further, to gather qualitative reactions about various FOPL designs, as the last part of the focus group discussions, participants were asked about NSS FOPL and shown images of three beverages (soft drink, chocolate milk, powdered juice) (see online supplementary material, Supplemental 2), with three different hypothetical NSS label formats. Top-selling brands for each beverage type were selected using Euromonitor 2022 data^(26)^ and contained both added sugars and NSS (see online supplementary material, Supplemental 2). Because beverages high in added sugar are already required to carry a FOPL in Brazil^(12)^, the beverages presented the mandatory FOPL for added sugars along with one of the three NSS FOPL (see online supplementary material, Supplemental 2).

**Figure 1.**
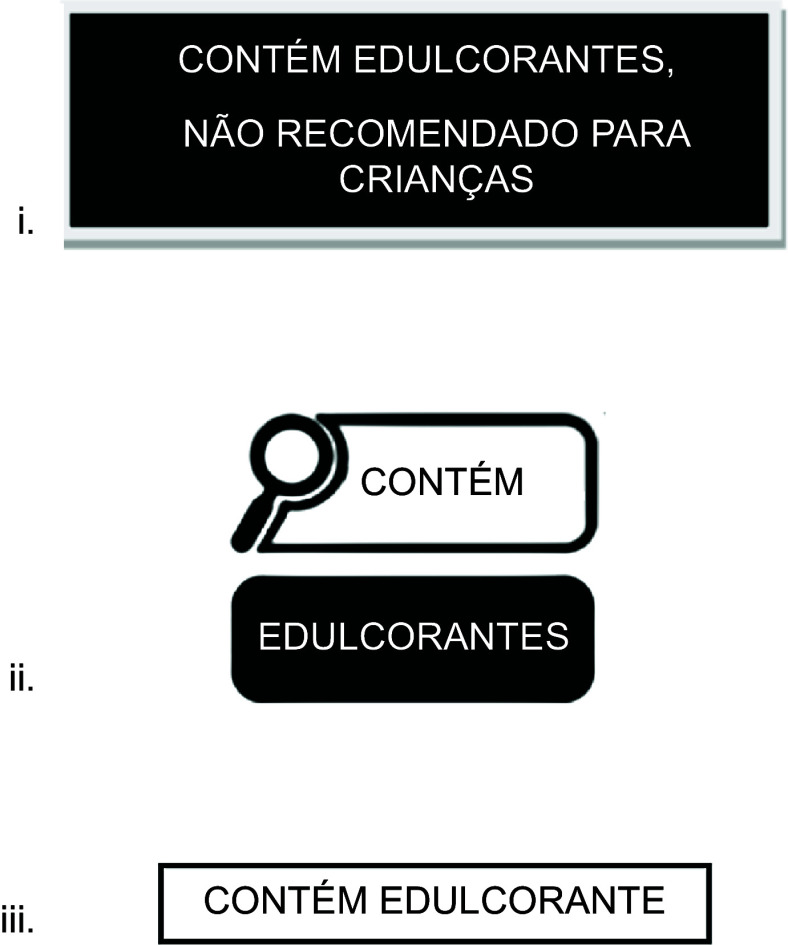
Non-sugar sweetener front-of-package labels (FOPL) shown to study participants.

The three NSS FOPL designed for this study included Label 1: a FOPL analogous to the one implemented in Mexico^(27)^ that read, ‘contains non-sugar sweeteners – not recommended for children’ (‘*contém edulcorantes – não recomendado para crianças’*); Label 2: a magnifying glass alike the FOPL implemented in Brazil^(12)^ that read, ‘contains non-sugar sweeteners’ (‘*contém edulcorantes*’); and Label 3: a phrase added to the bottom of the package that read, ‘contains non-sugar sweeteners’ (‘*contém edulcorantes*’) resembling information on flavouring content in Brazil. It is stipulated that products containing flavouring agents include a statement indicating their presence on the packaging; for example, flavoured beverages must display phrases such as ‘*Contém aromatizante*’ (‘Contains flavouring’) near the list of ingredients or elsewhere on the package (Figure 1, see online supplementary material, Supplemental 2).

The NSS FOPL used in the study were carefully selected to reflect labelling systems with existing regulatory frameworks^(27)^, which were either already implemented or familiar in Brazil^(12)^. One FOPL that read ‘not recommended for children’ label was based on the NSS FOPL already implemented in Mexico. Another NSS FOPL was based on the magnifying glass already used for other nutrients of concern in Brazil, which is a recognisable format for Brazilian consumers. The third one was modelled after Brazil’s current labelling requirements for flavouring^(28)^.

### Quantitative component: non-sugar sweetener identification and label survey

After the focus group discussions, participants completed a brief survey developed by the research team (see online supplementary material, Supplemental 3). The survey was intended to complement the qualitative insights gained from the focus group discussions. Administering the survey after the focus group discussion ensured that participants’ responses in the focus group were not influenced or constrained by the structure or content of the survey, allowing for more open and transparent dialogue during the focus groups.

The survey adapted measures that were taken from previous research on FOPL^(29)^ and focused on how different FOPL designs influence parents’ understanding and purchasing intentions. The survey (see online supplementary material, Supplemental 3) included questions to assess participants’ familiarity with beverage products that contain NSS, their ability to identify beverage products with and without NSS, their ability to identify NSS from lists of ingredients found on beverage packages and their perceptions and reactions to different designed FOPL for NSS on beverage packages. To capture participants’ views of FOPL, the survey included structured questions assessing health concerns, attention to the label, influence on purchasing decisions and perceived acceptance by other parents, based on previous surveys about acceptability and understanding of FOPL used in Latin America^(30)^ and other countries^(31)^, and adapting for NSS FOPL and purchase intention to children. Survey questions were in Portuguese.

In the survey, participants were shown images of five beverage products, including a yogurt drink, unsweetened whole milk, chocolate milk, a soft drink and powdered juice, two of which (chocolate milk and powdered juice) contained NSS (see online supplementary material, Supplemental 3). The products shown were beverages widely consumed by children in Brazil, based on the largest portion of the market share, provided by Euromonitor 2022 data^(26)^ for each beverage category. The images were used to assess participants’ ability to identify NSS only by looking only at the front-of-package of the product packages. Participants then viewed beverages’ list of ingredients (without the images) to assess their ability to identify NSS. Before completing the survey, participants received instructions on how to answer the questions. They were guided to mark the images they believed contained NSS and to highlight any terms in the list of ingredients they thought indicated NSS.

Then, participants were asked to respond to a series of structured, closed-ended questions about the three NSS FOPL they saw during the focus group discussion. In the survey, the labels were presented without embedding them in product packaging to isolate the effect of the labels on parental perceptions without other packaging elements influencing responses. Specifically, participants were asked to indicate the extent to which they worried about health effects, whether they thought their child would like to consume a beverage with the label, whether the label would discourage them from purchasing the product for their child, whether it caught their attention and how acceptable they believed it would be for other parents. Responses were provided on a four-point scale: ‘Not at all’, ‘A little’, ‘Very’ and ‘Quite a lot’ for the first four questions and ‘Not acceptable’, ‘Slightly acceptable’, ‘Acceptable’ and ‘Very acceptable’ for the last question. In addition, participants were asked two binary (Yes/No) questions: whether they liked the label as a tool for identifying NSS and whether they found the label easy to understand.

### Data analyses

Demographic characteristics and BFQ responses were summarised using descriptive analyses (frequencies and percentages). The BFQ was used to identify the percentage of children who consumed products containing NSS (tea, coffee or coffee with milk with added NSS, artificially sweetened beverages and powdered milk) per week and per day over the last month.

#### Qualitative component: focus group discussions

Recordings of the focus group discussions were transcribed and translated bi-directionally (a process of translating a document into another language (English) and then back into the original language (Portuguese) by a native speaker. The translations were checked by two additional individuals fluent in both Portuguese and English. The translated transcripts were then coded independently by two researchers who had been trained in qualitative coding using a data management software called Dedoose™. A combined deductive and inductive approach was employed to address both pre-existing topics derived from the focus group discussion guide, while also allowing themes to emerge from the data^(32)^. The analysis followed the Thematic Framework Analysis method to ensure the study not only explored predetermined areas of interest but also captured new and unexpected insights from participating parents^(33)^.

Following a step-by-step process^(34)^, the research team began by familiarising themselves with the data through reviewing focus group transcripts, followed by deductive coding based on research questions from the semi-structured guide. They then applied inductive coding to identify new themes, developing a codebook to ensure consistent application of both deductive and inductive codes. The codebook was refined throughout the coding process, based on regular discussions among the research team to ensure consistency in the coding approach.

After coding was completed, discrepancies between the two primary coders were reviewed and discussed with a third research team member, who was trained in qualitative coding and analysis. Once a final consensus was reached, representative quotes were selected to illustrate key themes and were chosen to reflect a range of participant experiences and provide clear, illustrative examples of the study findings.

Reflexivity and positionality were acknowledged as important considerations in the qualitative research process, given the potential influence of the researchers’ knowledge of the evidence on the health impacts of NSS and commitment to public health nutrition that may have shaped perspectives. To address this, regular discussions were conducted to recognise and reduce potential biases. Efforts were made to approach the study of NSS and FOPL objectively. For example, a clear coding framework was developed to ensure consistency in data analysis, and all methodological decisions were carefully documented to maintain transparency and rigour in data collection and analysis.

#### Quantitative component: exploratory survey

Quantitative data from the survey were analysed using descriptive statistics, including percentages and frequencies. The proportion of participants who reported familiarity with each beverage and those who regularly purchased each one for their children was calculated. Additionally, the percentage of participants who correctly identified which beverages contained NSS (i.e. chocolate milk and powdered juice) was determined. The proportion of participants who mistakenly identified beverages without NSS as containing NSS and the proportion of participants who correctly recognised NSS-related terms in the list of ingredients were also calculated. Likewise, the proportion of participants who incorrectly identified non-NSS terms as NSS was computed.

For the NSS FOPL component of the survey, responses were also analysed using descriptive statistics. For questions with Likert scale response options, responses at the high end (e.g. ‘Very’ and ‘Quite a lot’) of the scale were combined to calculate the proportion of participants who agreed with the statements, both overall and by school type.

All data were analysed using Stata/MP 18.0.

## Results

A total of seven focus groups were conducted with support from five schools in scheduling the sessions. Among them, four focus groups were with parents (*n* 23) of children aged 2–5 years old and three focus groups with parents of children aged 6–11 years old (*n* 17), totalling forty participants. Each focus group had between three and eight participants and lasted 45–65 min. Thematic saturation was reached after completing the seven focus groups, as the themes identified were robust and no new codes emerged.

### Individual characteristics and beverage frequency questionnaire

Participants’ mean age was 37·9 years old and their children were, on average, 6 years old. Most participants were female (*n* 35, 87·5 %), white (*n* 16, 40 %) and completed college (*n* 22, 55 %). In addition, most of the participants had their children in public school (*n* 27, 67·5 %) (Table 1).

**Table 1:** Sociodemographic characteristics of the participants (*n* 40)

	*n*	%
Sex		
Male	5	12·5
Female	35	87·5
Race		
Black	16	40·0
White	24	60·0
Educational attainment		
Upper elementary	1	2·5
High school	15	37·5
Trade school	2	5·0
Undergraduate degree	8	20·0
Graduate degree	14	35·0
Child’s school		
Private	13	32·5
Public	27	67·5

Approximately one-third (*n* 14, 35 %) of participants reported that their child consumed at least one beverage containing NSS (tea, coffee or coffee with milk with added NSS; artificially sweetened beverages; and powdered juice) per week over the past month, while 17 % (*n* 7) indicated that their child consumed beverages with NSS at least one time per day (results not shown).

### Focus group

Four overarching themes were identified (Table 2). First, participants described a preference for providing natural beverages to their children and aimed to avoid artificial ingredients whenever possible. Many participants expressed a strong desire to offer fresh, homemade juices or beverages with minimal additives, viewing them as healthier options. However, they also acknowledged that industrialised beverages are often more convenient and preferred by their children. Participating parents of children in private schools reported providing homemade drinks, whereas those in public schools described wanting to provide homemade drinks but faced challenges in doing so. Participants recruited from public schools cited barriers such as time constraints, cost and accessibility, which often led them to rely on packaged beverages, such as powdered juices, despite their preference for natural options.

**Table 2:** Overarching themes and subthemes identified in the focus group discussions

Theme #1. Parents want to provide their children with products that are natural and avoid those that contain artificial ingredients	
Subtheme: Preference for natural products for their children.	*‘My children have a lot of water. […] When she goes out it’s juice or Gatorade sometimes. Juice, we try 100 % natural but sometimes powdered juice’. (public school)* *‘At home, it’s water, juice, which I prefer natural’. (private school)*
Subtheme: Preference for juice fruit/real fruit.	*‘So normally when we go out, that’s when he drinks natural juice [that I prefer], because at home I have this difficulty. Fruits, things like that, are more expensive. So when we go out, it’s easier to get one’. (public school)* *‘When I go to buy the boxed juice, we always pay attention to the percentage of the juice itself’. (private school)*
Subtheme: Preference for products without/with less food additives.	*‘Sometimes she drinks milk, tea; she really likes tea. We buy lemongrass, chamomile, and make tea with sugar because no one deserves it with a sweetener. And then we have a lot of tea at home’. (public school)* *‘We try to buy those that have less quantities of colouring and sweeteners’. (private school)*
Subtheme: Preference for fewer ingredients.	*‘For example, yogurt. What are the selection criteria? I look at the number of ingredients. So, some brands only have dairy and milk yeast’. (public school)* *‘I base myself on that, the one with the least amount of ingredients’. (private school)*
Theme #2. Parents are confused about NSS	
Subtheme: Confusion with sugar.	*‘So, is this a type of sugar? A coloured sugar?’ (public school)* *‘In my mind, NSS could also be a sugar concentrate’. (private school)*
Subtheme: Confusion with food colouring.	*‘I think everything is a combination. Sugar and colour to modify the food, something like that’. (public school)* *‘It’s called “edulcorantes”, it looks like it’s a food colouring’. (private school)*
Subtheme: Difficulty identifying NSS in products.	*‘I don’t have the slightest idea [in which beverages NSS are]’. (public school)* *‘I think everything should have it, right? Juice, yogurt’. (private school)*
Subtheme: Confusion about whether NSS are healthy	*‘There’s not much to say because first, I don’t know what it is and secondly, it’s going to replace one thing that’s going to do harm with another’. (public school)* *‘I don’t know [if NSS are healthy], I use it. I don’t know what harm it causes, so I use it’. (private school)*
Theme #3. Parents want to avoid providing products high in sugar but do not think children should consume non-sugar sweeteners	
Subtheme: Avoid providing products high in sugar to children.	*‘We are very cautious at home about sugar’. (public school)* *‘If I look and see that there is sugar, I don’t offer it’. (private school)*
Subtheme: Worry about providing NSS.	*‘My concern is the future. And I think the ideal would be not to [give NSS to children]’. (public school)* *‘It’s addictive, right? If you introduce sweeteners to children, their organism ends up becoming addicted [to sweetness], right?’ (private school)*
Subtheme: Perceive NSS as helpful in specific situations.	*‘Maybe thinking about the restriction on calories, yes [it can help], but then I think it impacts other issues’. (public school)* *‘I think that can be necessary because there are children who have diabetes, I think they need to have a diet restricted in sugar. I don’t know any other way. Or refrain from drinking, or perhaps use a low-calorie natural sweetener, I don’t know. Because if it is for a special diet, the name itself says’. (private school)*
Theme #4. Parents notice the recently implemented FOPL and find FOPL NSS labels helpful for deciding what beverages to provide to their children	
Subtheme: Parents notice the new FOPL label in Brazil.	*‘Now it’s clearly stated on the packaging: it contains a high sugar content. It gets easier then’. (public school)* *‘I saw this label [the FOPL for added sugar]’. (private school)*
Subtheme: A label for NSS is helpful to identify NSS in products.	*‘It would be useful to choose, right, for you to have the option of choosing whether to give this to your child, to take it home or to your family’. (public school)* *‘In my case, I think it would be useful to identify things that contain NSS and that are not obvious, that do not say diet, light, for example. It’s not an obvious thing, a beverage, for example, juice. So, for these foods, I think it would be decisive for me when choosing’. (private school)*
Subtheme: The phrase ‘not recommended for children’ is particularly impactful.	*‘I wouldn’t purchase it because it’s not suitable for my child’. (public school)* *‘It is saying “not recommended for children”. In addition to getting straight to the point, the child reading is also important’. (private school)*

Second, there was confusion surrounding the term ‘*edulcorantes*’, with many participants indicating uncertainty as to whether the term referred to a type of sugar or a food colouring. While some recognised certain products containing NSS, such as liquid tabletop sweeteners, most were unaware of which beverages contained NSS or whether these ingredients were suitable for children (Table 2). Participants from private schools generally demonstrated a higher level of awareness, as some were able to name specific NSS types, such as aspartame and sucralose, whereas those from public schools were less familiar with these ingredients. However, regardless of school type, there was pervasive uncertainty about the presence of NSS in beverages, their role in children’s diets and the potential health implications of their consumption.

Participants’ concerns stemmed from a broader sense of distrust and uncertainty surrounding artificial ingredients. This scepticism was closely tied to their preference for ‘natural’ products, as many participants expressed caution towards ingredients perceived as artificial or chemically modified. As a result, they adopted a precautionary approach, opting to limit their children’s NSS consumption rather than taking risks with unfamiliar ingredients. Despite this general aversion to artificial ingredients, some participants, mostly parents of children from private schools, explained that certain types of NSS, such as stevia, might be more natural than others like aspartame and questioned whether NSS were derived from natural sources or if they were synthetic. Participants from both types of schools, however, explained that sugar was more familiar and therefore more acceptable than NSS. Others associated NSS with food colouring and were sceptical about their safety.

Third, participants were aware of the health risks associated with excessive sugar consumption in children, particularly to obesity and diabetes. However, they did not perceive NSS as a suitable alternative to sugar. Participants generally viewed NSS as appropriate only in specific cases, such as for individuals managing diabetes or weight loss, rather than for routine consumption by children. Although participants were uncertain about the potential risks of NSS, some expressed concerns about health effects, particularly regarding a potential link to cancer, which were largely based on information encountered in the media. While participants acknowledged the importance of reducing sugar intake and found it relatively easy to identify high-sugar products, they struggled to recognise products containing NSS.

Fourth, participants were familiar with the FOPL currently implemented in Brazil for products high in sugar, sodium and fat and noted they had seen these labels on various products in grocery stores and recognised them as a helpful tool for making informed food choices. When discussing a potential FOPL for NSS, they expressed strong support for a label modelled after Mexico’s FOPL for NSS, which explicitly stated that products with NSS were ‘not recommended for children’. Participants found this FOPL particularly helpful because it clearly signalled that the product was not for children, even if they were unsure about the definition of ‘*edulcorantes*’. Across all focus groups, regardless of school type, there was broad agreement that this FOPL would provide straightforward information that would support their ability to make healthier choices for their children. Additionally, in some discussions, participants pointed out the potential contradiction of having an FOPL stating that a product was ‘not recommended for children’ on packaging that featured extensive advertising directed at children (Table 2).

In addition to the overarching themes detailed above, participants also described interconnected factors that influenced the beverages they provided to their children and often described navigating trade-offs between affordability, convenience and perceived health impacts when selecting beverages for their children. Participants acknowledged that their own habits and their children’s exposure to marketing played a significant role in beverage selection, often reinforcing preferences for certain products despite describing concerns about NSS. Additionally, participants noted a contradiction between the presence of a warning label, such as ‘not recommended for children’, and packaging that includes colours, images or branding clearly directed at children. For instance, some participants expressed economic constraints as impacting their purchasing decisions, and others noted that marketing strategies strongly influenced children’s beverage preferences. Additionally, some participants reported that their children preferred sweet flavours, making it difficult to transition away from sweet products even when they had reservations about their healthfulness (Table 2).

### Non-sugar sweetener identification and label survey

When shown only the front-of-pack of five beverages (of which two contained NSS) without the ingredients list or the hypothetical NSS FOPL, the majority (87 %) of participants indicated familiarity with the beverages shown, and approximately one-third (33 %) reported regularly purchasing them for their child. Of the NSS-containing beverages (chocolate milk and powdered juice), 85 % of participants correctly identified them as containing NSS. However, most participants mistakenly indicated that the yogurt drink (62 %) and the soft drink (55 %) also contained NSS (Table 3). When shown the list of ingredients for these same five beverage products, 82 % of participants correctly identified at least one NSS-related term. However, the same percentage (82 %) mistakenly identified at least one non-NSS term as being an NSS. The terms most mistaken as NSS were sugar syrup (42 %), caramel (32 %), colouring (32 %) and flavouring (30 %) (see online supplementary material, Supplemental 4).

**Table 3:** Participants’ familiarity, purchasing and identification of non-sugar sweeteners (NSS) in beverages (*n* 40)

	Familiarity (%)	Purchase (%)	Perception that it contains NSS (%)
Yogurt drink	97·5	55·0	62·5
Unsweetened whole milk	97·5	60·0	7·5
Chocolate milk	97·5	30·0	85·0
Soft drink	95·0	35·0	55·0
Powdered juice	87·5	37·5	85·0

When shown the hypothetical NSS FOPL (Table 4), most participants (82 %) expressed concern about the health effects of beverages with Label 1, and more than half indicated concern about the health effects of beverages with Label 2 (64 %) and Label 3 (54 %). When asked if they thought their child would like to consume a beverage with each label, only 28 % reported their children would want to consume the product with Labels 1 and 2. In terms of participants’ reported willingness to offer these beverages to their children, Label 1 was the most discouraging (79 %), with 90 % of participants reporting that the label was noticeable and 90 % indicating the label would be accepted by other parents. Label 1 was overall the most preferred, with 95 % of participants indicating that it was helpful to identify NSS, compared with the other two labels. Label 2 (64 %) was the second most preferred, and Label 3 (33 %) was the least preferred. Label 1 was also reported to be the easiest to understand (95 %), followed by Label 2 (54 %) and Label 3 (36 %) (Table 4).

**Table 4:** Participants’ perceptions of the three hypothetical non-sugar sweeteners front-of-package labels (*n* 40)

	Label 1						Label 2						Label 3					
	Total sample		Public school		Private school		Total sample		Public school		Private school		Total sample		Public school		Private school	
	%	95 % CI	%	95 % CI	%	95 % CI	%	95 % CI	%	95 % CI	%	95 % CI	%	95 % CI	%	95 % CI	%	95 % CI
Do you worry about the health effects of your children consuming a beverage with this label																		
Not at all/A little	17·9	8·6, 33·7	19·2	7·9, 39·9	15·4	3·3, 49·3	35·9	22·2, 52·4	46·2	27·6, 65·8	15·4	3·3, 49·3	46·2	30·9, 62·2	46·2	27·6, 65·8	46·2	20·3, 74·2
Very/Quite a lot	82·1	66·3, 91·4	80·8	60·1, 92·1	84·6	50·7, 96·7	64·1	47·6, 77·8	53·8	34·2, 72·4	84·6	50·7, 96·7	53·8	37·8, 69·1	53·8	34·2, 72·4	53·8	25·8, 79·7
Do you think your child would like to consume a beverage with this label?																		
Not at all/A little	71·8	55·3, 84	65·4	44·7, 81·5	84·6	50·7, 96·7	71·8	55·3, 84	65·4	44·7, 81·5	84·6	50·7, 96·7	64·1	47·6, 77·8	65·4	44·7, 81·5	61·5	31·6, 84·7
Very/Quite a lot	28·2	16, 44·7	34·6	18·5, 55·3	15·4	3·3, 49·3	28·2	16, 44·7	34·6	18·5, 55·3	15·4	3·3, 49·3	35·9	22·2, 52·4	34·6	18·5, 55·3	38·5	15·3, 68·4
Does this label discourage you from giving a beverage with this label to your child?																		
Not at all/A little	20·5	10·4, 36·5	26·9	12·9, 47·8	7·7	0·9, 44·6	46·2	30·9, 62·2	61·5	41·1, 78·6	15·4	3·3, 49·3	73·7	57, 85·5	76	54·6, 89·3	69·2	37·8, 89·3
Very/Quite a lot	79·5	63·5, 89·6	73·1	52·2, 87·1	92·3	55·4, 99·1	53·8	37·8, 69·1	38·5	21·4, 58·9	84·6	50·7, 96·7	26·3	14·5, 43	24	10·7, 45·4	30·8	10·7, 62·2
Does this label catch your attention?																		
Not at all/A little	10·3	3·8, 25	15·4	5·6, 35·8	0	0, 0	38·5	24·3, 54·9	50	30·8, 69·2	15·4	3·3, 49·3	79·5	63·5, 89·6	80·8	60·1, 92·1	76·9	44·3, 93·3
Very/Quite a lot	89·7	75, 96·2	84·6	64·2, 94·4	100	0, 0	61·5	45·1, 75·7	50	30·8, 69·2	84·6	50·7, 96·7	20·5	10·4, 36·5	19·2	7·9, 39·9	23·1	6·7, 55·7
To what extent do you believe that this label would be accepted by parents in general?																		
Not acceptable/Slightly acceptable	10·3	3·8, 25	11·5	3·6, 31·6	7·7	0·9, 44·6	28·2	16, 44·7	30·8	15·6, 51·6	23·1	6·7, 55·7	41	26·5, 57·3	42·3	24·5, 62·4	38·5	15·3, 68·4
Acceptable/Very acceptable	89·7	75, 96·2	88·5	68·4, 96·4	92·3	55·4, 99·1	71·6	55·3, 84	69·2	48·4, 84·4	76·9	44·3, 93·3	59	42·7, 73·5	57·7	37·6, 75·5	61·5	31·6, 84·7
Do you like this label to help you identify NSS? (%)																		
No	5·1	1·2, 19	0	0, 0	15·4	3·3, 49·3	35·9	22·2, 52·4	42·3	24·5, 62·4	23·1	6·7, 55·7	35·9	22·2, 52·4	42·3	24·5, 62·4	23·1	6·7, 55·7
Yes	94·9	81, 98·8	100	0, 0	84·6	50·7, 96·7	64·1	47·6, 77·8	57·7	10·1, 75·5	76·9	44·3, 93·3	64·1	47·6, 77·8	57·7	37·6, 102	76·9	44·3, 93·3
Is this label easy to understand? (%)																		
No	5·1	1·2, 19	3·8	0·5, 24·6	7·7	0·9, 44·6	46·2	30·9, 62·2	53·8	34·2, 72·4	30·8	10·7, 62·2	64·1	47·6, 77·8	73·1	52·2, 87·1	46·2	20·3, 74·2
Yes	94·9	81, 98·8	96·2	75·4, 99·5	92·3	55·4, 99·1	53·8	37·8, 69·1	46·2	27·6, 65·8	69·2	37·8, 89·3	35·9	22·2, 52·4	26·9	12·9, 47·8	53·8	25·8, 79·7

Label 1: a front-of-package label (FOPL) analogous to the one implemented in Mexico that read, ‘contains non-sugar sweeteners – not recommended for children’ (‘*contém edulcorantes – não recomendado para crianças*’); Label 2: a magnifying glass alike the FOPL implemented in Brazil that read, ‘contains non-sugar sweeteners’ (‘*contém edulcorantes*’); and Label 3: a phrase added to the bottom of the package that read, ‘contains non-sugar sweeteners’ (‘*contém edulcorantes*’) resembling information on flavouring content in Brazil.

When stratified by school type (public and private), there were few statistically significant differences. For Label 1, a divergence emerged in approval rates for its utility in identifying NSS beverages: public school participants reported unanimous endorsement (100 % ‘Yes’; CI: 0–0), contrasting with 84 % (CI: 501–97) in private schools. However, most comparisons, including health concerns, label discouragement and attention-catching, did not have statistically significant differences (Table 4).

## Discussion

The findings indicate that while the Brazilian parents were generally confused about ‘*edulcorantes*’ (the Portuguese term for NSS), they expressed concern about the health impacts of both sugar and NSS on their children. Overall, while some differences in beverage accessibility and NSS awareness were observed between public and private school parents in this study, their concerns and preferences for a NSS FOPL were largely aligned. Participants noted that an FOPL for NSS would assist in beverage selection, with a preference for labels explicitly stating, ‘contains non-sugar sweeteners – not recommended for children’, which discouraged providing NSS-containing beverages to children. Collectively, the focus group and survey results underscore both the difficulty in identifying NSS products and a strong preference for a clear FOPL that discourages NSS for children. Given the qualitative nature of this study, these findings are specific to this sample and should not be viewed as representative of all Brazilian parents.

In the focus group, participants expressed a desire to provide ‘natural’ products for their children and avoid artificial ingredients. The preference for ‘natural’ products is consistent with a larger global trend where claims of naturalness are equated with healthfulness^(35)^. In fact, the Brazilian Dietary Guidelines emphasise the consumption of natural and minimally processed foods while recommending avoidance of ultra-processed products, which may positively influence consumer awareness and dietary choices^(36)^. However, there is also the possibility of a ‘health halo’ effect associated with ‘natural’ or ‘low in’ claims, which can lead consumers to perceive ‘natural’ products as inherently safer or healthier, regardless of their actual nutritional content^(37)^. A recent study about consumer perceptions of NSS in yogurts showed that consumers perceived products labelled as natural as being healthier and more desirable, despite their nutritional content^(38)^. For example, in the aforementioned study about yogurt, consumers demonstrated a clear preference for the ‘naturally sweetened’ claim over a ‘reduced sugar’ claim (notably, the ‘naturally sweetened’ yogurt contained NSS, yet parents may perceive natural NSS such as stevia and monk fruit as healthier than ‘artificial’ NSS such as aspartame or sucralose, despite a lack of research directly comparing the metabolic and health effects of ‘natural’ *versus* ‘artificial’ NSS^(39)^. The lack of a clear regulatory definition for ‘natural’ adds to consumer confusion and allows for potentially misleading marketing, yet consumers are still willing to pay a premium for these products^(38)^.

A key difference that emerged between public and private school participants was related to accessibility and consumption habits. Participants from public schools often cited cost, time constraints and convenience as barriers to providing natural or homemade beverages for their children. This observation aligns with previous research indicating that socio-economic factors significantly influence beverage choices among parents. For instance, a study examining factors influencing parents’ provision of beverages to their children highlighted that while most parents are aware of the negative health effects of frequent sweet beverage intake among children, sociodemographic factors pose barriers to limiting their children’s consumption^(40)^. As a result, they reported relying more on packaged beverages, such as powdered juices, despite a stated preference for natural products.

Participants from private schools also generally demonstrated a slightly higher level of awareness about NSS, with some being able to name specific NSS from tabletop sweeteners. However, across both groups, there was substantial confusion about the term ‘*edulcorantes*’, with many in both groups mistakenly associating the term with ‘coloured sugar’ or food colouring. This may reflect a lack of clear communication about NSS in product labelling and public health messaging. The phonetic similarity between the Portuguese terms ‘*edulcorantes*’ (NSS) and ‘*corantes*’ (colourings) may exacerbate this confusion and underscores the importance of ensuring that parents can distinguish between different types of ingredients and make informed dietary choices for their children.

While most participants could correctly identify beverages containing NSS when completing the survey, there was still confusion, with some mistakenly identifying products without NSS as containing them as well. Since the term ‘*edulcorantes*’ led to confusion, further research could help identify terminology that is more intuitive and accessible for parents. Investigating how consumers interpret different labelling terms and testing alternative wording could enhance clarity in both FOPL and the list of ingredients. Beyond labelling, this confusion indicates there may be an opportunity to enhance public awareness about NSS and other food additives. Initiatives aimed at improving food literacy could help clarify the differences between various sweeteners, address common misconceptions and provide practical guidance on interpreting product labels. By fostering a clearer understanding of food ingredients and their functions, such efforts could support more informed decision-making for parents.

Participants in both public and private schools expressed a strong desire to reduce their children’s sugar consumption, reflecting the growing public health awareness of the risks associated with excessive sugar intake^(41)^. While participants reported avoiding sugary products, they did not view NSS as an alternative. Uncertainty about the long-term health effects of NSS among participants, even without fully understanding their impact, may be amplified by mixed messages in public discourse. This lack of clear and consistent information may lead parents to avoid providing NSS to their children. However, participants recognised that NSS could be beneficial in specific cases, such as for children with diabetes or those needing a calorie-restricted diet.

Participants also acknowledged the significant role that children’s preferences play in determining what beverages they purchase. This finding aligns with research showing that marketing to children has a profound influence on their food choices^(42)^. This finding also highlights a broader challenge in public health communication: when FOPL appear alongside packaging that visually appeals to and is directed to children, the intended health message may be undermined or even contradicted. Such inconsistencies between labelling and marketing strategies can lead to confusion among consumers and could potentially reduce the credibility or perceived urgency of the warning itself. Policies that include both FOPL and labelling restrictions can address this disconnect and potentially improve the effectiveness of FOPL. For example, Chile’s food labelling and advertising law prohibits child-directed marketing, such as cartoon characters and games, on products carrying warning labels, reinforcing the message that these products are not appropriate for children and promoting healthier choices^(43)^.

The widespread presence of NSS in products marketed to children^(17)^, combined with participants’ confusion on where NSS are found (as revealed in the focus group data) and the terms used to list them in ingredient labels (as highlighted by the survey), underscores the potential utility of an NSS FOPL to mitigate this confusion. FOPL are among the recommended food environment strategies for childhood obesity in Latin America^(44)^, and mounting evidence demonstrates that they are indeed effective in influencing perceptions of product healthfulness and purchasing decisions^(45)^. Evidence from Mexico demonstrates that an NSS FOPL increased awareness of the population about the presence of NSS in food products^(46)^. Given that participants were aware of the FOPL recently implemented for nutrients of public health concern in Brazil and found FOPL helpful in guiding their purchasing decisions, it is likely that an NSS FOPL would be similarly well-received.

During focus group discussions and the survey, participants, irrespective of whether they were from public or private schools, preferred the rectangular NSS label modelled after Mexico’s FOPL, which stated ‘contains non-sugar sweeteners – not recommended for children’. They found the rectangular shape more alarming and the direct message particularly effective in discouraging purchases of NSS products for their children. In fact, public school participants unanimously endorsed this label. This preference may reflect the label’s direct messaging and rectangular format, which participants described as ‘alarming’ and effective in discouraging purchases of NSS products for children, a finding consistent with public health communication principles emphasising clarity and directness to influence consumer behaviour, especially regarding children’s health decisions^(45)^.

Several limitations of this study should be considered, along with the steps taken to address them. First, the relatively small sample size (*n* 40) and the recruitment of participants from only two cities in São Paulo limit the generalisability of the findings to the broader Brazilian population, as perceptions may differ across regions with diverse sociodemographic and cultural contexts. However, participants were recruited from both public and private schools to elicit a diverse range of perspectives. In addition, participants might have provided socially desirable responses during focus group discussions; however, to mitigate this, participants were assured of confidentiality, and the semi-structured guide included neutral, open-ended questions to encourage honest and varied responses. It is also possible that the consumption of NSS among children was underestimated because the BFQ included only beverages highly likely to contain NSS, while other beverages containing NSS may not have been captured. However, we conservatively opted to focus on beverages most representative of NSS consumption patterns in Brazil. Finally, the perceptions captured in the survey may have been influenced by having already participated in a focus group discussion. However, this was an intentional design choice, as the primary aim of the study was to gain deeper insights from the focus groups, with the survey serving as a complementary quantitative component.

Key strengths of the study include its qualitative-driven mixed-methods embedded design, which provided a comprehensive understanding of Brazilian parents’ perceptions of NSS. By combining focus group discussions with a survey, we collected both in-depth qualitative insights and measurable quantitative data. The study addresses a timely research gap, relevant to policymakers in Brazil and other Latin American countries, and the comparison of different FOPL designs offers actionable information for regulatory agencies. Including parents of younger (2–5) and older (6–11) children further strengthens the study, as similar results across these age groups demonstrate a consistent preference for clear labelling that discourages NSS for children.

Although most parents of school-aged children in Brazil, both from public and private schools included in this study, were unclear about the meaning of ‘*edulcorantes*’ (NSS), they expressed concerns about the potential health risks of both sugar and NSS in children’s diets. Participants showed a preference for a FOPL that clearly stated, ‘contains non-sugar sweeteners – not recommended for children’, and found it effective in discouraging them from purchasing such products for their children. Overall, the findings of this study underscored the challenges parents face in identifying NSS in products and emphasised that an FOPL could help mitigate this confusion and support parents in making more informed dietary choices for their children, regardless of socio-economic background.

## Supporting information

Grilo et al. supplementary material Grilo et al. supplementary material

## Supplementary material

For supplementary material accompanying this paper visit https://doi.org/10.1017/S1368980025101146.
