# Supplementary material for: Perceptions of non-sugar sweeteners and front-of-package labels among parents of preschool and school-aged children in Brazil
Source: Public Health Nutr. 2025 Sep 19;28(1):e170. doi: 10.1017/S1368980025101146 (PMC12723420; doi:10.1017/S1368980025101146)
Supplement: Grilo et al. supplementary material [file S1368980025101146sup001.docx]

**Supplementary Files**

**Supplementary 1.** Focus group discussion guide

1. What type of beverage do you give to the child?

2. What factors influence the types of beverages you offer to the child?

3. What characteristics do you look for in beverages when choosing what to give to the child? (nutrients, ingredients)

a) Are there certain nutrients/ingredients you want to provide to the child? Why or why not?

b) Are there certain nutrients/ingredients you try to avoid for the child? Why or why not?

4. Do you usually provide these beverages to the child: powdered juice, soft drinks, milk drinks, unsweetened or diet/light/zero yogurt? Why or why not?

5. Do you know what a non-sugar sweetener (NSS) is?

Explanation of what a NSS is: Food additives approved by ANVISA in certain quantities, with one of their main functions being to provide a sweet taste to foods and beverages without adding calories or with very low addition. They are also called “sugar substitutes,” “sweeteners,” or “dietary sweeteners.” Commonly known as table sweeteners (liquid or powder), they are found in many diet foods and drinks, for example, but also appear in various other foods and beverages. Example: saccharin.

6. Where do you think NSS are found?

7. What types of beverages do you think contain NSS?

a) Can you recall any others?

8. What is your opinion on NSS in beverages?

9. What do you think about giving NSS to the child?

a) What do you think about the child consuming NSS instead of added sugars?

b) How much do you think they help the child reduce sugar or calorie intake or control weight?

c) How much do you see them as healthy or unhealthy for the child to consume?

10. Now, changing the subject a bit, I’ll ask a few questions related to the new nutritional label implemented in October of last year in Brazil that requires a front-of-package label for added sugars, saturated fat, and sodium when a food or drink contains high amounts of these nutrients. [show image]

a) Have you noticed this new labeling?

b) Does having this information on the front of the package help you make decisions about foods and beverages to offer the child?

11. How useful do you think it would be to have clearer warnings about the presence of sweeteners on the packaging?

a) For example, what do you think of a warning label – for example, “contains non-sugar sweeteners, not recommended for children”? [show example]

b) And if it were together with the new magnifying glass symbol? [show example]

c) Or having a phrase that says “contains non-sugar sweeteners” on the front of the package? [show example]

12. Do you think having these types of NSS labels would affect your choice of which beverages to buy for your children? Why or why not?

13. Is there anything you would like to add about NSS and their consumption among children? And is there any additional question you thought I would ask but didn’t?

**Supplementary 2.** Front-of-package labels (FOPL) on three beverages.

**Beverages with FOPL 1:**

**
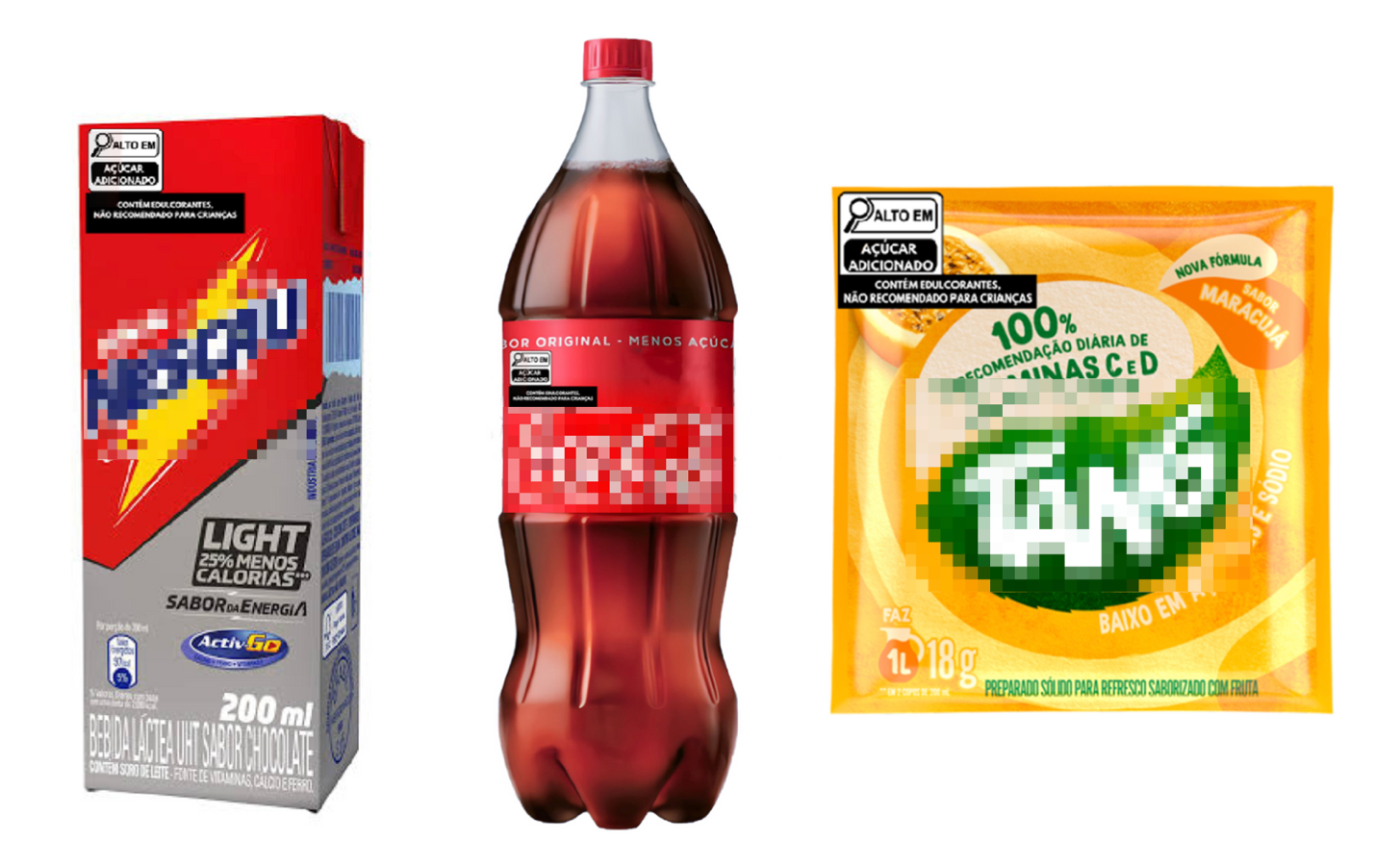
**

**Beverages with FOPL 2:**

**
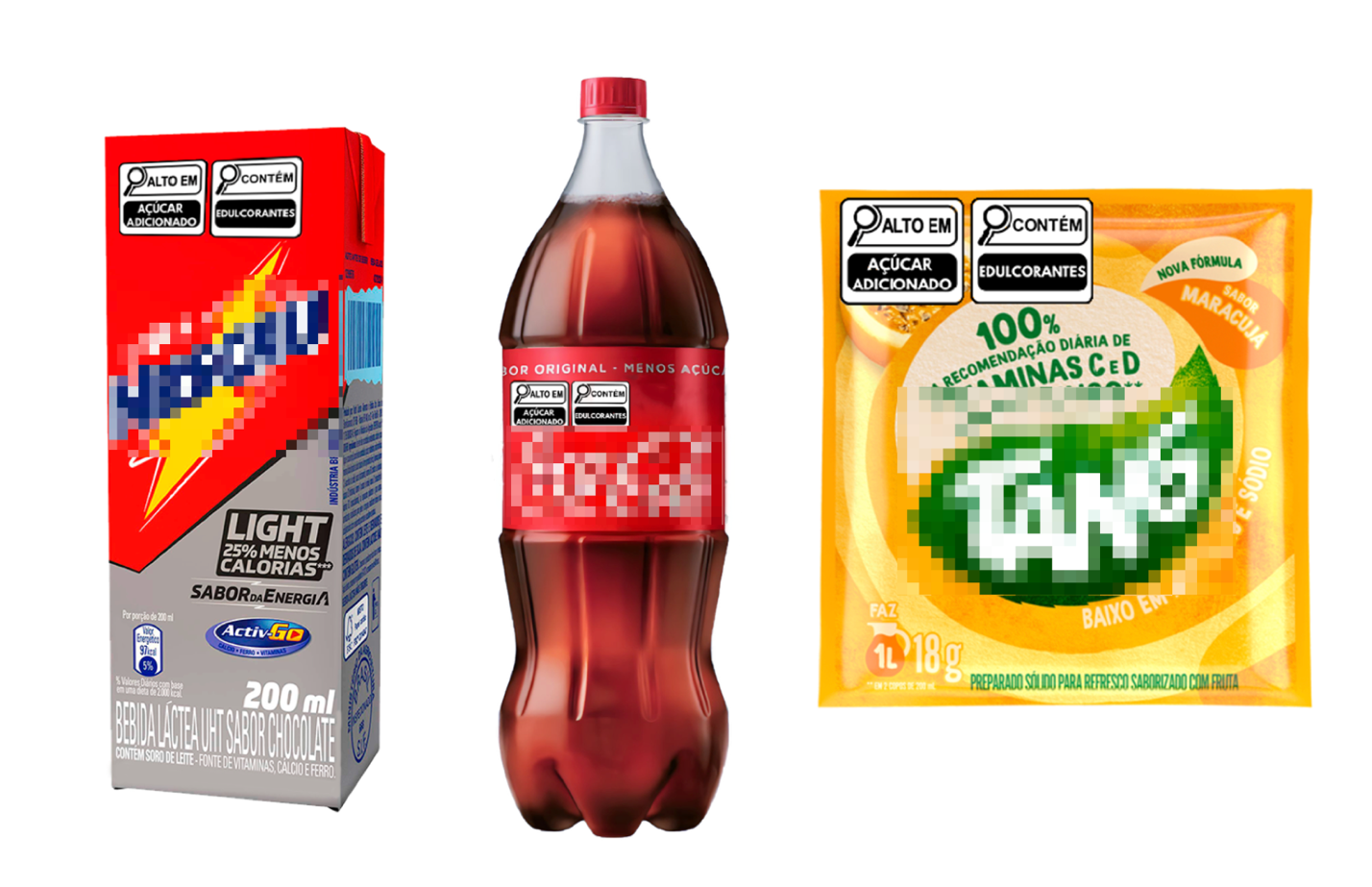
**

**Beverages with FOPL 3:**

**
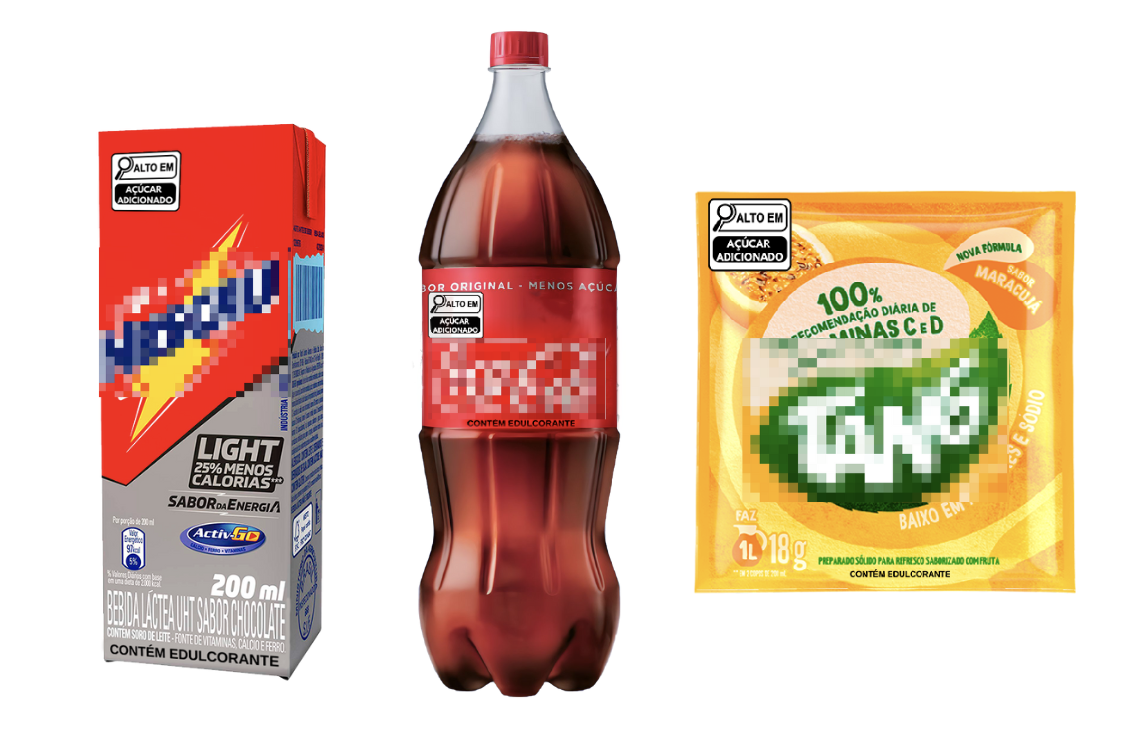
**

Label 1: a front-of-package label (FOPL) analogous to the one implemented in Mexico that read, “contains non-sugar sweeteners - not recommended for children” (“*contém edulcorantes – não recomendado para crianças”*), Label 2: a magnifying glass analogous to the FOPL implemented in Brazil but that read, “contains non-sugar sweeteners” (“*contém edulcorantes*”), and Label 3: a phrase added to the bottom of the package that read, “contains non-sugar sweeteners” (“*contém edulcorantes*”)) resembling how the presence of flavorings is disclosed on product packages in Brazil

**Supplementary 3.** Survey to assess parent’s ability to identify non-sugar sweeteners (NSS) and perceived message effectiveness of the hypothetical NSS front-of-package nutrition labels.

1. Below are several examples of different products. We want to explore your understanding of beverages sold in the market.

a. Please circle which of these products you are familiar with (consider these brands or similar ones).


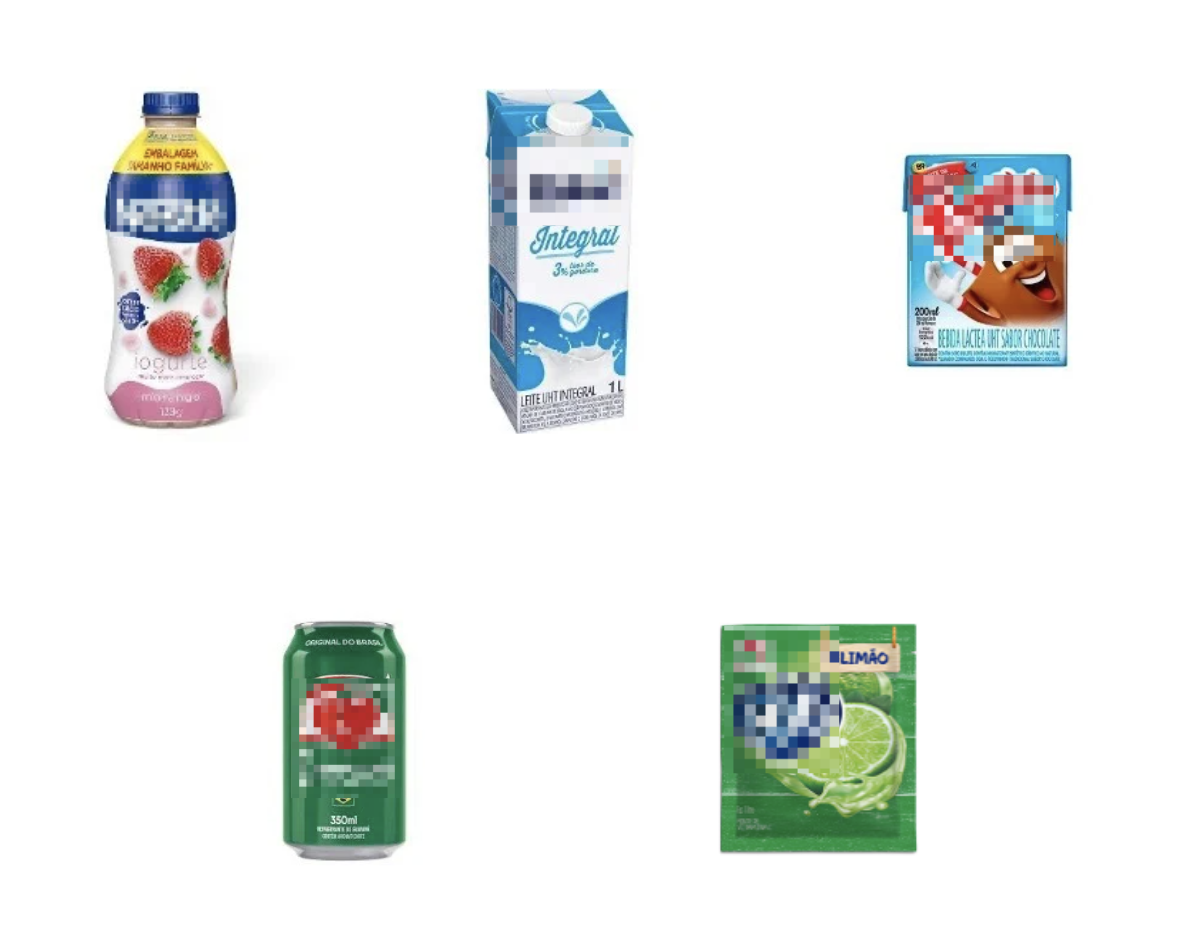


b. Please circle which of these products you purchase for the child (consider these brands or similar ones).


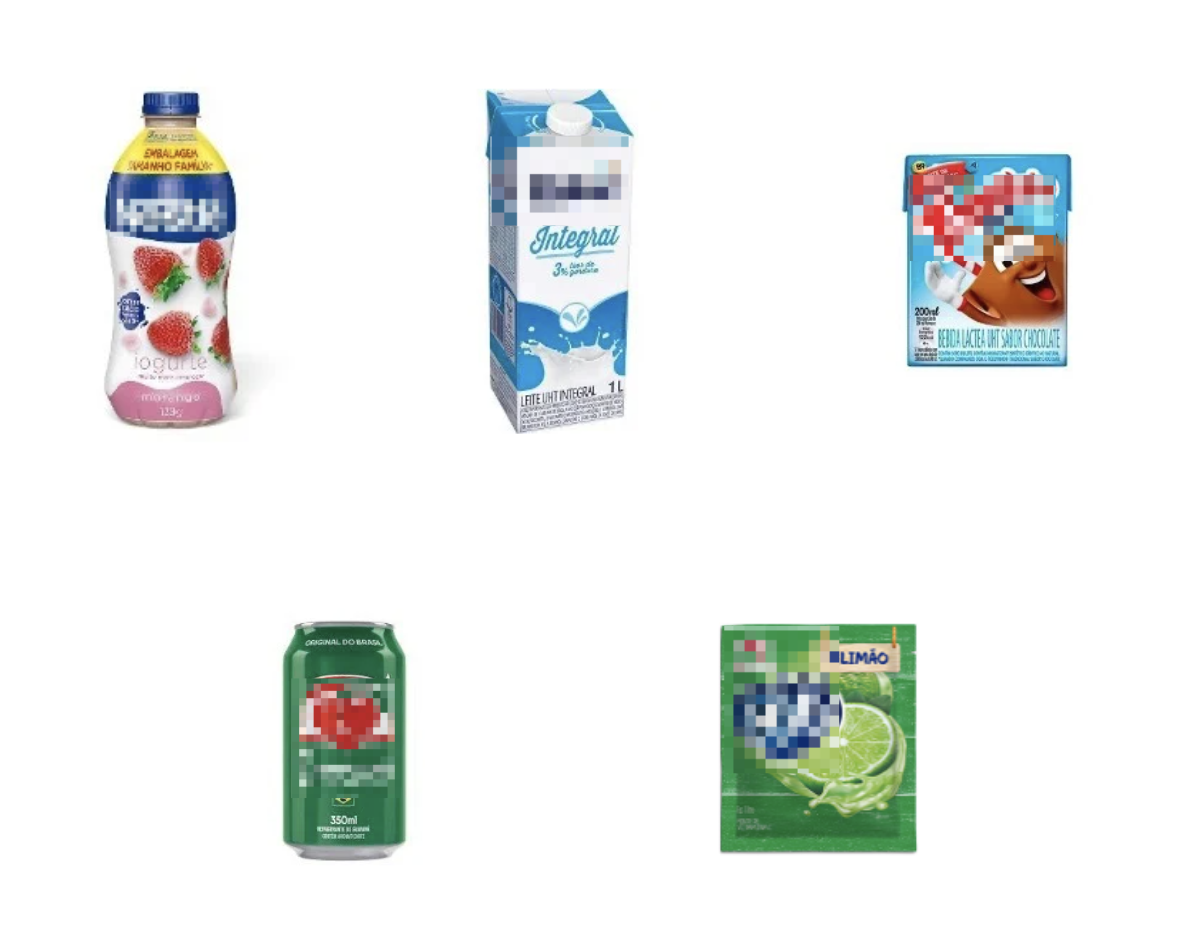


c. Now, ONLY REGARDING THE PRODUCTS AND BRANDS LISTED BELOW.
Please circle which of these products you think contain NON-SUGAR SWEETENERS.


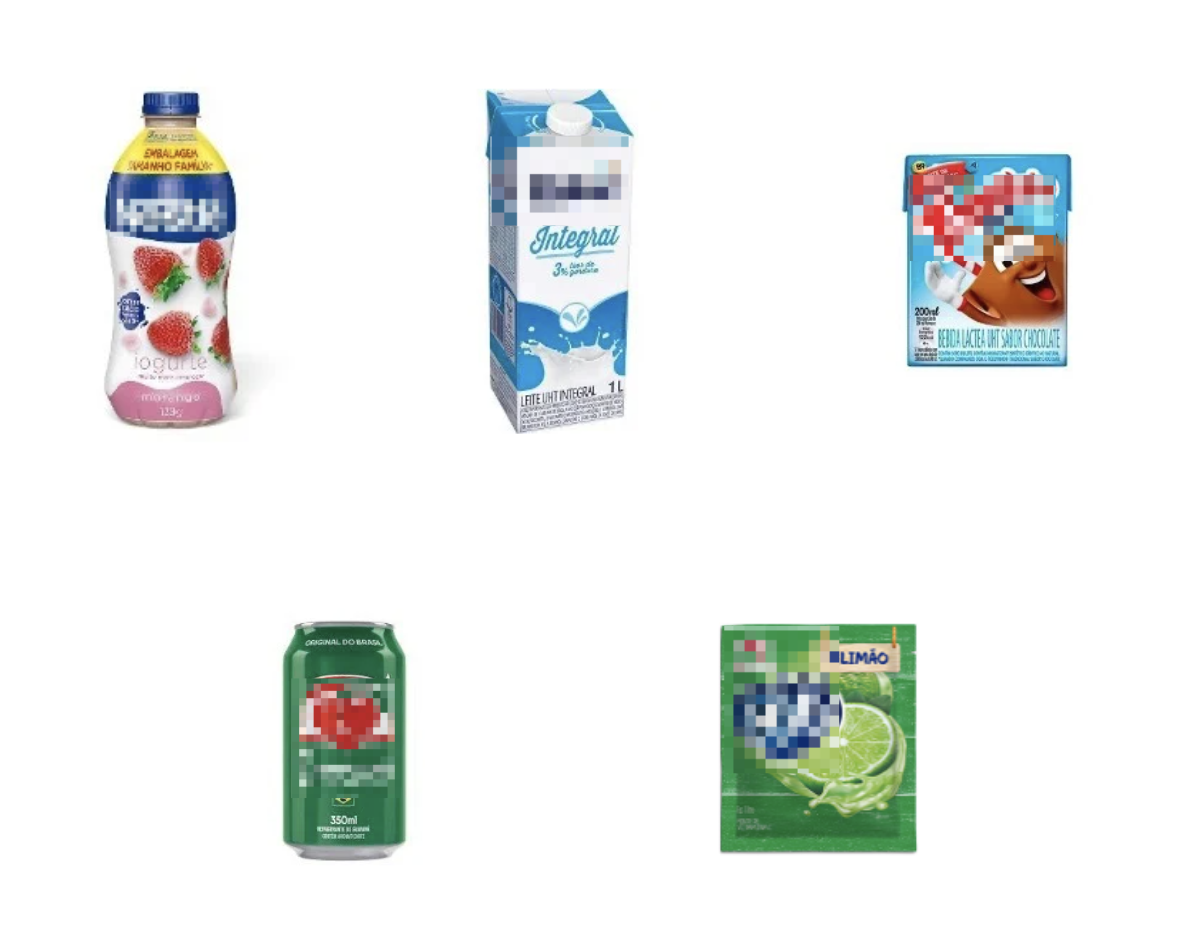


2. Still about NON-SUGAR SWEETENERS.
Below are some examples of ingredient lists that you probably have seen on the back of beverage packaging. Please circle which ingredients you believe are non-sugar sweeteners.

- - 1. **Ingredientes:** leite fermentado parcialmente desnatado e/ou leite pasteurizado integral, preparado de morango (agua, xarope de açúcar, polpa de morango, amido modificado, açúcar, fosfato tricalcico, aromatizantes, acidulante acido cítrico, espessante goma guar, corante natural carmim, conservador sorbato de potássio), soro de leite e/ou soro de leite reconstituído, xarope de açúcar, soro de leite em pó e fermento lácteo.
    2. **Ingredientes**: Leite integral e estabilizantes citrato de sódio, trifosfato de sódio, monofosfato monossódico e difosfato dissódico.
    3. **Ingredientes:** leite desnatado reconstituído, cacau hidratado, soro de leite reconstituído, leite integral reconstituído, açúcar, extrato de malte, vitaminas (C, A e Ácido Fólico), espessantes: goma gelana, carboximetilcelulose sódico e goma guar, estabilizante citrato de sódio, edulcorantes acesulfame de potássio e sucralose emulsificante lecitina de soja e aromatizante.
    4. **Ingredientes:** água gaseificada, açúcar, extrato de guaraná, acidulante ácido cítrico, conservadores: benzoato de sódio e sorbato de potássio, aromatizante e corante caramelo.
    5. **Ingredientes:** açúcar, suco de limão desidratado, maltodextrina, vitamina c (ácido ascórbico), acidulante ácido cítrico, aromatizante aroma natural de limão, antiumectante fosfato tricálcico, edulcorantes artificiais (por 100 ml: aspartame: 24,6 mg, acesulfame de potássio: 10,1 mg e advantame: 0,01 mg), regulador de acidez, citrato de sódio, corante inorgânico dióxido de titânio e corante artificial tartrazina.

3. Now, let's talk about NON-SUGAR SWEETENER LABELS.
Circle one of the answer options for each question.

**Label 1.**

**CONTÉM EDULCORANTES,**

**NÃO RECOMENDADO PARA CRIANÇAS**

Do you worry about the health effects on the child when consuming a beverage with this label?
1: Not at all
2: A little
3: Very
4: Quite a lot

Do you think the child would like to consume a beverage with this label?
1: Not at all
2: A little
3: Very
4: Quite a lot

Does this label discourage you from wanting your child to consume this product?
1: Not at all
2: A little
3: Very
4: Quite a lot

Does this label catch your attention?
1: Not at all
2: A little
3: Very
4: Quite a lot

To what extent do you believe this label would be accepted by parents and guardians in general?
1: Not acceptable
2: Slightly acceptable
3: Acceptable
4: Very acceptable

Do you like this label to help you identify NSS? (%)
1: Yes
2: No

Is this label easy to understand?
1: Yes
2: No

Now this second label:

**Label 2.**


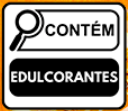


(Same questions as for Label 1)

Lastly, this third label:

**Label 3.**


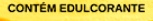


(Same questions as for Label 1)

**Supplementary 4.** Parents’ identification of non-sugar sweeteners (NSS) in the list of ingredient of five beverages

|  | Parents that identified the ingredient as NSS (%) |
| --- | --- |
| ***List of ingredients 1*** |  |
| Fermented Partially Skimmed Milk And/Or Pasteurized Whole Milk | 10.0 |
| Strawberry Preparation | 10.0 |
| Water | 2.5 |
| Sugar Syrup | 20.0 |
| Strawberry Pulp | 2.5 |
| Modified Starch | 12.5 |
| Sugar | 15.0 |
| Tricalcium Phosphate | 12.5 |
| Flavorings | 20.0 |
| Citric Acidulant | 15.0 |
| Guar Gum Thickener | 5.0 |
| Natural Carmine Coloring | 10.0 |
| Preservative | 10.0 |
| Potassium Sorbate | 10.0 |
| Whey And/Or Reconstituted Whey | 5.0 |
| Sugar Syrup | 42.5 |
| Whey Powder | 2.5 |
| Milk Yeast | 7.5 |
|  |  |
| ***List of ingredients 2*** |  |
| Whole Milk | 2.5 |
| Stabilizers | 7.5 |
| Sodium Citrate | 2.5 |
| Sodium Triphosphate | 7.5 |
| Monosodium Monophosphate | 2.5 |
| Disodium Diphosphate | 2.5 |
|  |  |
| ***List of ingredients 3*** |  |
| Reconstituted Skimmed Milk | 2.5 |
| Hydrated Cocoa | 0.0 |
| Reconstituted Whey | 5.0 |
| Reconstituted Whole Milk | 7.5 |
| Sugar | 12.5 |
| Malt Extract | 5.0 |
| Vitamin C | 0.0 |
| Vitamin A | 0.0 |
| Folic Acid | 0.0 |
| Thickeners | 5.0 |
| Gellan Gum | 12.5 |
| Sodium Carboxymethylcellulose | 7.5 |
| Guar Gum | 7.5 |
| Sodium Citrate Stabilizer | 2.5 |
| Sweeteners* | 75.0 |
| Acesulfame Potassium* | 60.0 |
| Sucralose* | 37.5 |
| Emulsifier | 20.0 |
| Soy Lecithin | 15.0 |
| Flavoring | 20.0 |
|  |  |
| ***List of ingredients 4*** |  |
| Carbonated Water | 2.5 |
| Sugar | 12.5 |
| Guarana Extract | 17.5 |
| Citric Acidulant | 20.0 |
| Preservatives | 12.5 |
| Sodium Benzoate | 5.0 |
| Potassium Sorbate | 5.0 |
| Flavoring | 30.0 |
| Caramel | 32.5 |
| Coloring | 32.5 |
|  |  |
| ***List of ingredients 5*** |  |
| Sugar | 7.5 |
| Dehydrated Lemon Juice | 2.5 |
| Maltodextrin | 17.5 |
| Vitamin C (Ascorbic Acid) | 2.5 |
| Citric | 12.5 |
| Acidulant | 17.5 |
| Natural Lemon Flavoring | 20.0 |
| Tricalcium Phosphate | 5.0 |
| Anti-Wetting Agent | 5.0 |
| Artificial Sweeteners* | 67.5 |
| Aspartame* | 35.0 |
| Acesulfame Potassium* | 35.0 |
| Advantame* | 35.0 |
| Acidity Regulator | 5.0 |
| Sodium Citrate | 7.5 |
| Inorganic Coloring | 25.0 |
| Titanium Dioxide | 12.5 |
| Artificial Coloring | 27.5 |
| Tartrazine | 27.5 |

*Terms for non-sugar sweeteners
